# Dose Effects of Oxaliplatin on Persistent and Transient Na^+^ Conductances and the Development of Neurotoxicity

**DOI:** 10.1371/journal.pone.0018469

**Published:** 2011-04-08

**Authors:** Susanna B. Park, Cindy S-Y. Lin, Arun V. Krishnan, David Goldstein, Michael L. Friedlander, Matthew C. Kiernan

**Affiliations:** 1 Prince of Wales Clinical School, University of New South Wales, Sydney, New South Wales, Australia; 2 Neuroscience Research Australia, University of New South Wales, Sydney, New South Wales, Australia; 3 School of Medical Sciences, University of New South Wales, Sydney, New South Wales, Australia; 4 Department of Medical Oncology, Prince of Wales Hospital, Sydney, New South Wales, Australia; Dalhousie University, Canada

## Abstract

**Background:**

Oxaliplatin, a platinum-based chemotherapy utilised in the treatment of colorectal cancer, produces two forms of neurotoxicity- acute sensorimotor neuropathic symptoms and a dose-limiting chronic sensory neuropathy. Given that a Na^+^ channelopathy has been proposed as the mechanism underlying acute oxaliplatin-induced neuropathy, the present study aimed to determine specific mechanisms of Na^+^ channel dysfunction.

**Methodology/Principal Findings:**

Specifically the function of transient and persistent Na^+^ currents were followed during treatment and were investigated in relation to oxaliplatin dose level. Eighteen patients were assessed before and after a single oxaliplatin infusion with motor and sensory axonal excitability studies performed on the median nerve at the wrist. While refractoriness (associated with Na^+^ channel inactivation) was significantly altered post-oxaliplatin infusion in both motor (Pre: 31.7±6.4%; Post: 68.8±14.5%; P≤.001) and sensory axons (Pre: 31.4±5.4%; Post: 21.4±5.5%; P<.05), strength-duration time constant (marker of persistent Na^+^ conductances) was not significantly altered post-infusion (Motor Pre: 0.395±0.01 ms; Post: 0.394±0.02 ms; NS; Sensory Pre:0.544±0.03 ms; Post: 0.535±0.05 ms; NS). However, changes in strength-duration time constant were significantly correlated with changes in refractoriness in motor and sensory axons (Motor correlation coefficient = −.65; P<.05; Sensory correlation coefficient = .67; *P*<.05).

**Conclusions/Significance:**

It is concluded that the predominant effect of acute oxaliplatin exposure in human motor and sensory axons is mediated through changes in transient rather than persistent Na^+^ conductances. These findings are likely to have implications for the design and trial of neuroprotective strategies.

## Introduction

Oxaliplatin is a third generation platinum-based chemotherapy effective in the treatment of colorectal cancer [1], [2], [3]. Oxaliplatin treatment is limited by prominent neurotoxicity which develops immediately following infusion and accumulates across treatment [4]. Immediately following infusion, acute neuropathic symptoms develop in the majority of patients, consisting of cold-induced paresthesia, fasciculations and cramps which typically resolve within a week following infusion [5], [6]. With increasing cumulative dose, a chronic sensory neuropathy develops, leading to long-lasting functional disability [1], [4].

Recent experimental studies across a variety of models have suggested that oxaliplatin modulates axonal voltage-gated Na^+^ channels, specifically by slowing Na^+^ channel inactivation kinetics [7]–[9], shifting the voltage dependence of inactivation to more negative membrane potentials [10], or reducing Na^+^ current [10], [11]. However, there remains no consensus about the mechanisms underlying the development of an acute “Na^+^ channelopathy” in oxaliplatin treated patients, with *in vitro* studies inconsistent to date concerning the relative involvement of transient and persistent Na^+^ conductances [9], [11].

In previous studies, our group has identified striking changes in Na^+^ channel related parameters [12]–[15], however there has been no assessment of the relative contributions of Na^+^ conductance subtypes in both motor and sensory axons in the development of acute oxaliplatin-induced neurotoxicity. As Na^+^ channel modulation remains a potential neuroprotective strategy for oxaliplatin-induced neurotoxicity, it is important to investigate the specific types of Na^+^ conductances targeted by oxaliplatin. The present study utilised novel functional and specialised neurophysiological approaches to assess the effects of acute oxaliplatin administration on markers of axonal transient and persistent Na^+^ conductances and determined the dose-response relationship of oxaliplatin on Na^+^ currents *in vivo*.

## Methods

### Participants

Eighteen patients treated with oxaliplatin for colorectal cancer were prospectively assessed before and immediately following oxaliplatin infusion. Three of these patients were included in a prior study [15]. Patients received standard oxaliplatin containing treatment regimens with initial oxaliplatin doses ranging from 85–130 mg/m^2^ given every 2 to 3 weeks for a total of 6–12 treatment cycles. Oxaliplatin (FOLFOX 4 regimen: 85 mg/m^2^, FOLFOX 6 regimen: 100 mg/m^2^) was infused every 2 weeks in conjunction with leucovorin (200 mg/m^2^) and followed by bolus 5-fluorouracil (5-FU; 400 mg/m^2^). For the next 48 hours, a continuous infusion of 5-FU (600 mg/m^2^) was given, followed by leucovorin (200 mg/m^2^) and 5-FU bolus (400 mg/m^2^) on the 2^nd^ day [2], [16]. In patients receiving XELOX regimens, oxaliplatin (130 mg/m^2^) was given intravenously every 3 weeks with oral capecitabine (1000 mg/m^2^) twice daily for 2 weeks [17].

### Ethics

The study was approved by the South Eastern Area Health Service (Eastern Section) Human Research Ethics Committee and University of New South Wales Human Research Ethics Committee. Participants provided written informed consent in accordance with the declaration of Helsinki.

### Study Design and Objectives

Each patient was assessed once immediately prior to oxaliplatin infusion and once immediately following the same infusion to compare the changes pre- and post-infusion.

Patients were tested in treatment cycles from the 2^nd^ treatment to the 6^th^ treatment, corresponding a cumulative dose range of 200–650 mg/m^2^ oxaliplatin. For analysis, patients were divided into groups based on cumulative oxaliplatin dose (less than or greater than 400 mg/m^2^) as prior studies highlighted the changes in acute excitability parameters after this dose level [14], [15].

### Clinical Neurotoxicity Grading Scales

To assess the extent of acute neuropathic symptoms, the Oxaliplatin-Specific Neurotoxicity Scale (OSNS) was utilized with the following grades: Grade 1 – dysesthesia or paresthesia that completely regresses before the next cycle of therapy; Grade 2 – dysesthesia or paresthesia persisting between courses of therapy; and Grade 3 - dysesthesia or paresthesia causing functional impairment [18]. In addition the Neuropathy Sensory Subscale of the National Cancer Institute (NCI) Common Toxicity Criteria for Adverse Events Scale (Version 3) was utilized with the following grading system: Grade 1 (Mild) – loss of deep tendon reflexes or paresthesia not interfering with function; Grade 2 (Moderate) – sensory alteration or paresthesia interfering with function but not activities of daily living; Grade 3 (Severe) – sensory alteration or paresthesia interfering with activities of daily living; and Grade 4 – disabling [19].

### Neurophysiological Techniques

Multiple excitability studies were undertaken, utilising previously described protocols [20], [21] and threshold tracking Qtrac software (© Institute of Neurology, London, UK). Compound muscle action potentials (CMAPs) were recorded from the abductor pollicis brevis (APB) muscle with the reference electrode 4 cm distally. Compound sensory action potentials (CSAPs) were recorded from digit 2. Stimulation was applied via non-polarizable electrodes at the median nerve at the wrist, with the reference electrode placed 10 cm proximally, using an isolated linear bipolar constant-current stimulator (DS5, Digitimer Ltd., Welwyn Garden City, UK). Temperature at the site of stimulation was monitored and maintained greater than 32°C.

Stimulus-response curves were recorded by increasing the stimulus intensity until the maximal compound response amplitude was obtained. The stimulus threshold was defined as the stimulus intensity (mA) required to produce a compound response 50% of maximal amplitude. The stimulus-response slope was derived from the range between 25% and 75% of maximal amplitude [21]. Strength-duration time constant (SDTC) and rheobase were determined according to Weiss' Law, utilising the relationship between stimulus charge and stimulus duration [22], [23], as a marker of persistent Na^+^ channel activity [24]. In addition, latent addition protocols were utilised to assess the threshold increase at 0.2 ms, a marker of nodal persistent Na^+^ currents as SDTC measurements may be affected by other factors [24]. Brief 60 µs stimuli were utilised, with a conditioning stimuli set to −90% of threshold and the conditioning-test interval changed between 0.02 and 0.5 ms. Analysis was completed as in [24], [25]. The recovery cycle of excitability was assessed utilizing a paired pulse protocol, with a supramaximal stimulus followed by a test stimulus at intervals varying between 2.5 and 200 ms. Initially following conduction of an impulse, it becomes more difficult to generate a subsequent impulse due to the recovery of inactivation of transient Na^+^ channels [26], [27]. Refractoriness was calculated as the percentage increase in threshold at an interstimulus interval of 2.5 ms [26], [27]. The relative refractory period (RRP) was determined as the first intercept on the x-axis in the recovery cycle curve, corresponding to the interstimulus interval (ms) when threshold change was zero [20], [26]. Threshold electrotonus was assessed utilising 100 ms subthreshold polarizing currents (40% of threshold) in both hyperpolarizing and depolarizing directions [27], with threshold reduction assessed between 90 and 100 ms of polarizing current in both hyperpolarizing and depolarizing directions.

### Statistical Methods

All statistics were performed in SPSS (Version 18, SPSS Inc., Chicago, US). Results were expressed as mean ± standard error of the mean and statistical significance was defined as P≤0.05. Recordings were paired for each patient pre- and post-infusion. Wilcoxon sign rank tests (two-tailed) were utilised to compare paired recordings pre- and post-oxaliplatin within individual patients. Mann Whitney U tests (two-tailed) were used to compare findings within groups divided by dose level. Single oxaliplatin dose was defined as the oxaliplatin dose infused at the time of testing. Cumulative oxaliplatin dose was calculated as the sum total oxaliplatin dose received in all treatment cycles including the tested cycle. Correlations were performed using Spearman's rank correlation coefficients.

## Results

### Clinical Features

All patients reported the development of acute neuropathic symptoms following oxaliplatin infusion (average single dose 91±5 mg/m^2^). The most commonly reported symptom was cold-induced paresthesia in distal upper limbs, lasting for 2 to 10 days post infusion. The majority of patients displayed mild neurotoxicity (89%), with an NCI score of 1 and an OSNS neurotoxicity score of 1, reflecting the resolution of acute neuropathic symptoms within two weeks. 11% of patients had an OSNS score of 2, reflecting the persistence of neuropathic symptoms for greater than two weeks. Further patient characteristics are detailed in Table 1.

**Table 1:** Clinical features and patient demographics.

Patient	Age (gender)	Single oxaliplatin dose (mg/m^2^)	Cumulative oxaliplatin dose (mg/m^2^)	Cycle tested	OSNS/NCI
1	61 M	–	–	4	1
2	25 M	85	255	3	1
3	48 F	100	200	2	1
4	60 M	85	340	4	1
5	59 M	85	340	4	1
6	41 M	85	255	3	1
7	36 M	85	340	4	1
8	62 F	100	200	2	1
9	82 M	100	252	3	1
10	61 M	80	280	3	1
11	60 M	85	340	4	1
12	35 F	130	260	2	1
13	60 F	66	415	6	1
14	59 M	85	425	5	2
15	67 F	85	510	6	2
16	69 M	100	600	6	1
17	46 M	130	650	5	1
18	61 F	53	497	6	1

### Modulation of Na^+^ Channel Function with Oxaliplatin Infusion

**Figure 1:**
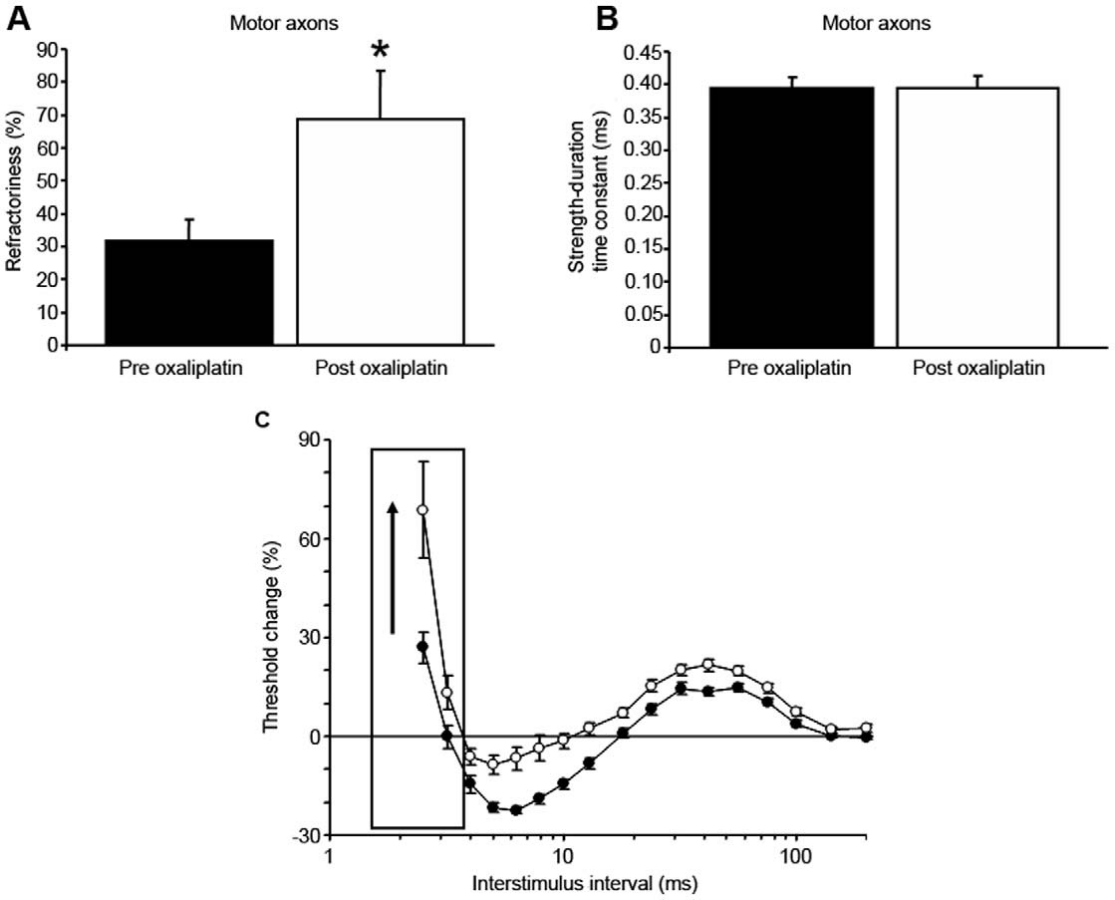
Changes in Na^+^ channel associated parameters post-oxaliplatin in motor axons. A) Refractoriness pre (black bars) and post (white bars) single oxaliplatin infusion demonstrating significant change post-oxaliplatin in motor axons (Refractoriness Pre: 31.7±6.4%; Post 68.8±14.5%; *P*≤.001). B) Strength-duration time constant pre (black bars) and post (white bars) oxaliplatin infusion, demonstrating unchanged results following oxaliplatin treatment (Pre: 0.395±0.01 ms; Post: 0.394±0.02 ms; NS). C) Inset diagram of the changes in the recovery cycle of excitability pre- (black circles) and post-oxaliplatin infusion (white circles) for all patients, with error bars representing standard error of the mean and refractoriness outlined by a box and an arrow demonstrating direction of change.

Immediately following oxaliplatin infusion, refractoriness was significantly increased in motor axons (Refractoriness Pre: 31.7±6.4%; Post 68.8±14.5%; *P*≤.001; Figure 1A; C), consistent with previous findings [13], [15]. Similarly, there was a trend towards increased RRP in motor axons (Pre: 3.4±0.2 ms; Post: 3.9±0.3 ms; NS). However in contrast, strength-duration time constant (SDTC) was unchanged following oxaliplatin treatment (Pre: 0.395±0.01 ms; Post: 0.394±0.02 ms; NS; Figure 1B). Accordingly, there were no significant changes in threshold change at 0.2 ms as assessed using latent addition (Pre: 11.2±1.2%; Post: 12.5±1.4%; NS). In addition, other stimulus-response and strength-duration properties, including rheobase (Pre: 3.2±0.2 mA; Post: 2.6±0.2 mA; NS), stimulus-response slope (Pre: 6.5±0.5; Post: 7.3±0.5; NS), and stimulus threshold (Pre: 4.4±0.3 mA; Post: 4.9±0.3 mA; NS) were also unchanged following oxaliplatin treatment. There were no major changes in threshold electrotonus in hyperpolarizing (Pre: −126.4±4.4%; Post: −125.8±3.8%; NS) or depolarizing directions (Pre: 45.7±0.9%; Post: 45.0±0.9%; NS), suggesting that axonal membrane potential was not significantly altered post-oxaliplatin treatment in motor axons. Maximal CMAP amplitude also remained unchanged post-oxaliplatin treatment (Pre: 6.7±0.7 mV; Post: 6.4±0.5 mV; NS). There were no significant differences in limb temperature pre- and post-oxaliplatin infusion (Pre: 32.1±0.2°C; Post: 3.2±0.2°C; NS). Of further relevance, all patients in the present study received intravenous chemotherapy through a central venous catheter rather than through a peripheral vein, and so the temperature of the testing arm was not affected by infusion-related issues.

**Figure 2:**
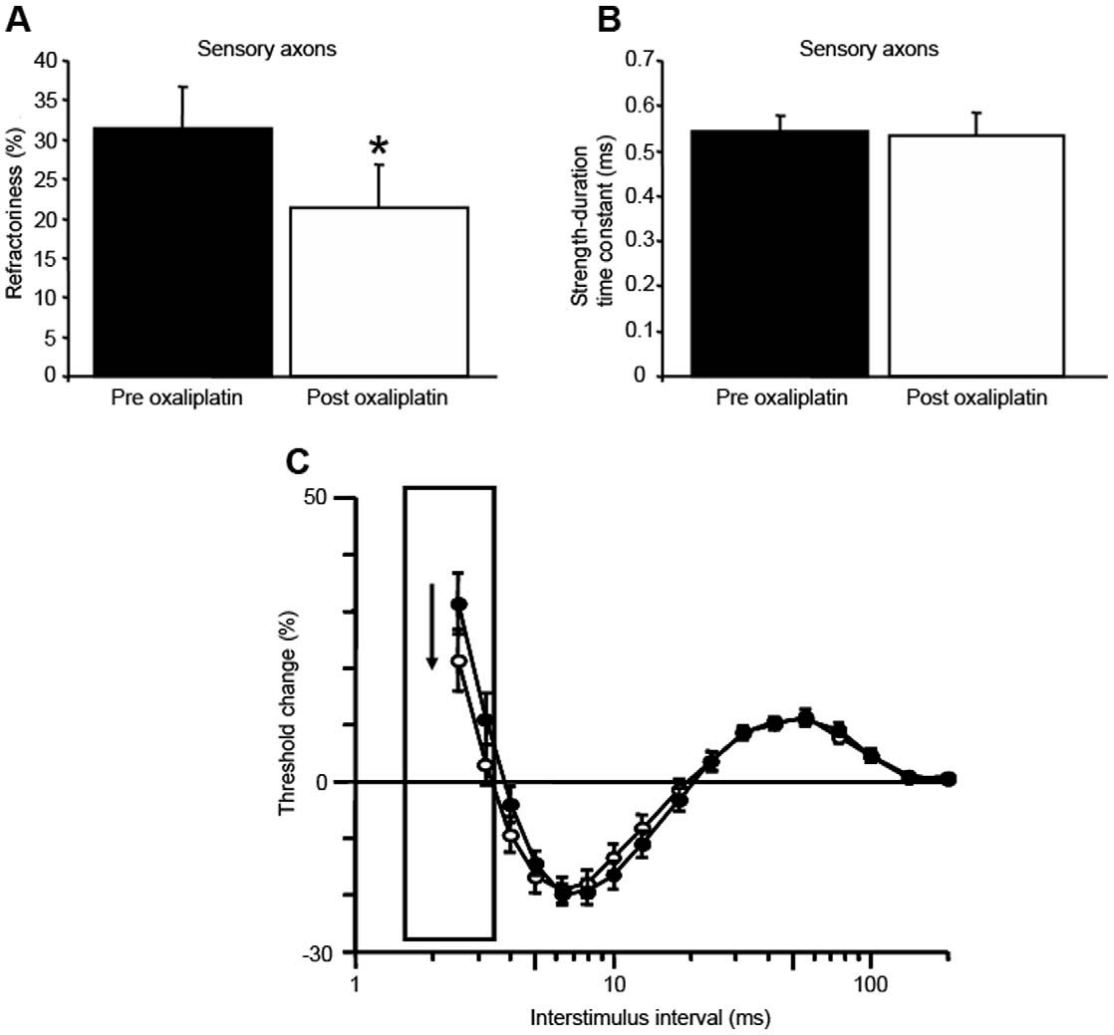
Changes in Na^+^ channel associated parameters post-oxaliplatin in sensory axons. A) Refractoriness pre (black bars) and post (white bars) oxaliplatin treatment (Pre: 31.4±5.4%; Post: 21.4±5.5%; *P*<.05), demonstrating significant reduction post-oxaliplatin in sensory axons. B) Strength-duration time constant pre (black bars) and post (white bars) treatment (Pre: 0.544±.03 ms; Post: 0.535±.05 ms; NS), with no significant effect post oxaliplatin treatment. C) Changes in the recovery cycle of excitability pre- (black circles) and post-oxaliplatin infusion (white circles) for all patients, with error bars representing standard error of the mean and refractoriness outlined by a box and an arrow demonstrating direction of change.

Conversely to these findings in motor axons, there was a significant reduction in refractoriness in sensory axons following oxaliplatin treatment (Pre: 31.4±5.4%; Post: 21.4±5.5%; *P*<.05; Figure 2A) in accordance with previous studies [14], [15]. In accordance with these changes, RRP was significantly reduced (Pre: 3.9±0.1 ms; Post: 3.4±0.2 ms; *P*<.05). However, there were no significant differences in SDTC (Pre: 0.544±.03 ms; Post: 0.535±.05 ms; NS; Figure 2B), rheobase (Pre: 2.3±0.4 mA; Post: 2.4±0.4 mA; NS) or stimulus threshold (Pre: 4.8±0.8 mA; Post: 5.2±0.9 mA; NS) following oxaliplatin treatment in sensory axons. As in motor axons, markers of axonal membrane potential remained unchanged (Threshold electrotonus hyperpolarizing Pre: −141.4±7.0%; Post: −142.8±8.7%; NS; TE depolarizing Pre: 51.2±1.6%; Post: 52.0±2.0%; NS). Maximal CSAP amplitude was unchanged post-oxaliplatin (Pre: 47.3±10.1 µV; Post: 44.3±10.7 µV; NS).

### Oxaliplatin Dose-Response Relationship

**Figure 3:**
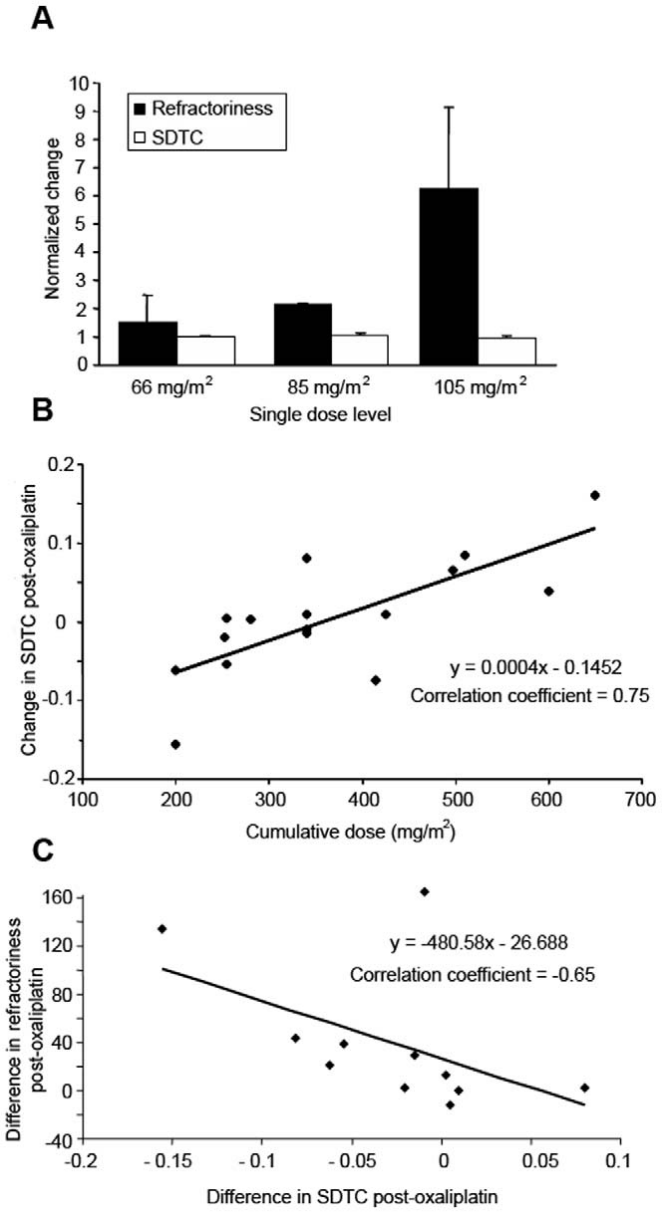
Relationship of Na^+^ channel-associated parameters with oxaliplatin dose level in motor axons. A) Relationship of Na^+^ channel-associated parameters with single oxaliplatin dose level in motor axons. Patients were divided into three single dose levels with means of 66, 85 and 105 mg/m^2^. Change in refractoriness was significantly correlated with increasing single dose (correlation coefficient = .80; *P*≤.001). B) Correlation of cumulative oxaliplatin dose with the change in strength-duration time constant post-oxaliplatin in motor axons, illustrating the association of higher cumulative doses with an increase in strength-duration time constant (Correlation coefficient = .75; *P*≤.001). C) Relationship of changes in refractoriness compared to changes in SDTC in motor axons. Change in refractoriness post-oxaliplatin was significantly associated with change in SDTC post-oxaliplatin (correlation coefficient = −.65; *P*<.05).

To determine the relationship of acute changes in Na^+^ channel-related parameters with oxaliplatin dose levels, comparisons were made across different dose levels. In motor axons, single oxaliplatin dose was significantly correlated with change in refractoriness following infusion (Correlation coefficient = .80; *P*≤.001; Figure 3A). While overall there was no significant changes in SDTC following oxaliplatin treatment, higher cumulative doses were associated with an increase in SDTC (Correlation coefficient = .75; P≤.001; Figure 3B). Similarly, there was an association between threshold change at 0.2 ms as assessed using latent addition and cumulative dose (Correlation coefficient = .66; *P*<.05). In patients assessed at a cumulative dose of less than 400 mg/m^2^, there was also a significant correlation between strength-duration properties and refractoriness. Change in refractoriness post-oxaliplatin was significantly correlated with change in SDTC (Correlation coefficient = −.65; *P*<.05; Figure 3C), suggesting a link between the mechanisms underlying changes in these parameters. Despite this, the overall sensitivity of refractoriness to change following oxaliplatin treatment was significantly greater than that of SDTC (Table 2).

**Table 2:** Sensitivity of excitability parameters to oxaliplatin treatment.

Parameter	Resting value	Post-oxaliplatin value	Percentage difference	P value
**Motor axons**				
Refractoriness (%)	35.7±8	75.5±21	213%	P<.05
Strength-duration time constant (ms)	0.395±.02	0.368±.02	−6%	P>.05
**Sensory axons**				
Refractoriness (%)	31.4±5	21.4±6	−65%	P<.05
Strength-duration time constant (ms)	0.544±.03	0.535±.05	−2%	P>.05

**Figure 4:**
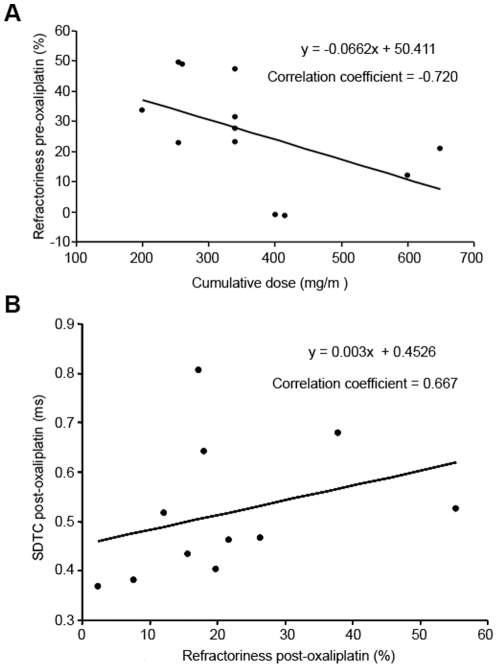
Relationship of Na^+^ channel-associated parameters with oxaliplatin dose level in sensory axons. A) Correlation of cumulative oxaliplatin dose with refractoriness pre-oxaliplatin infusion in sensory axons, demonstrating the development of chronic changes in sensory excitability with increasing dose (Correlation coefficient = −.72; *P*<.01). B) Relationship of refractoriness post-infusion and SDTC post-infusion in sensory axons (Correlation coefficient = .67; *P*<.05).

In sensory axons, the extent of change in pre-oxaliplatin refractoriness was determined by the cumulative oxaliplatin dose (Correlation coefficient = −.72; *P*<.01; Figure 4A), presumably reflecting the development of chronic changes in sensory axonal excitability with increasing dose. Therefore, patients with a cumulative dose of less than 400 mg/m^2^ were also assessed and accordingly, refractoriness post-oxaliplatin infusion was significantly correlated with SDTC post-oxaliplatin (Correlation coefficient = .67; *P*<.05), although again the extent of change in refractoriness far exceeded the change in SDTC (Table 2).

## Discussion

The present study represents the first assessment of the dose-response properties of acute and cumulative oxaliplatin exposure in human axons *in vivo*. Markers of transient and persistent Na^+^ conductances were investigated in patients receiving neurotoxic chemotherapy with oxaliplatin, to clarify existing *in vitro* data which suggested tremendous variability between experimental preparations. Importantly, the present study is the first to directly compare the relationship between oxaliplatin dose and transient and persistent Na^+^ conductances, in both motor and sensory axons. While there were significant changes in markers of Na^+^ channel inactivation in both motor and sensory axons post-oxaliplatin infusion, changes in markers of non-inactivating Na^+^ conductances were relatively minor. There were significant correlations between oxaliplatin dose level and Na^+^ channel-associated parameters, suggesting a dose-response relationship between Na^+^ channel dysfunction and oxaliplatin dose. Taken in total, the present findings suggest that acute oxaliplatin administration preferentially affects markers of transient Na^+^ conductances, at least in the clinical setting.

### Modulation of Transient and Persistent Na^+^ Conductances

In human peripheral nerves, both transient and persistent Na^+^ currents have been functionally identified [24], [28]–[30]. The transient Na^+^ current contributes an estimated 98% of the total nodal Na^+^ current [31]. While the persistent Na^+^ current accounts for only 1.0–2.5% of the total Na^+^ current [24], [32], it exerts a strong influence on membrane potential and axonal excitability [28], [31]. Strength-duration time constant (SDTC) provides a surrogate marker of persistent Na^+^ conductances active at threshold [23], [24], while refractoriness classically relates to inactivation of transient Na^+^ channels [27], [29]. While it has been proposed that transient and persistent currents both arise from Na_v_1.6 channels [33], [34], differences in channel gating and kinetic properties may have important functional consequences.

### Mechanisms of Oxaliplatin-induced Modulation of Na^+^ Channel Function

As in previous studies, a different pattern of excitability change was identified in sensory axons acutely following oxaliplatin administration [14], [15], with sensory changes qualitatively similar to the effects of the Na^+^ channel blocker tetrodotoxin [35]. Sensory and motor axons have significantly different biophysical properties [27] which may underlie differential expression of oxaliplatin-induced effects on recovery cycle parameters.

In motor axons, refractoriness depends on the transmission of impulses through the neuromuscular junction [27], [36]. The neuromuscular junction may be directly affected by oxaliplatin [8] or the ability of the neuromuscular junction to accurately transmit impulses may be affected as a by-product of spontaneous high frequency activity produced by oxaliplatin [5], [6]. Accordingly, the secondary effects of spontaneous activity on motor axonal excitability may mask the primary effect of acute oxaliplatin-induced neurotoxicity in motor axons, leading to a different observed pattern of changes in motor axons from sensory axons. Spontaneous activity in sensory axons could be expected to produce a different pattern of changes due to the lack of a neuromuscular junction. However, importantly, the extent of oxaliplatin-induced excitability abnormalities in both motor and sensory axons were correlated [14], suggesting that the factors underlying acute oxaliplatin-induced abnormalities in sensory and motor axons are linked.

*In vitro* studies have suggested that oxaliplatin modulates axonal Na^+^ channel inactivation properties via slowing of inactivation kinetics or changing the voltage dependence of activation or inactivation [7]–[10]. Studies in a motor neuron-like cell line suggesting that oxaliplatin primarily affected inactivation properties of the transient Na^+^ current, with no effect on the persistent current elicited by long ramp pulses [9]. Earlier studies in an insect experimental preparation had conversely suggested that oxaliplatin preferentially affected the persistent Na^+^ current [11]. However, there are known differences between the responses of insect Na^+^ channels and vertebrate Na^+^ channels to tetrodotoxin and other toxins which may underlie these apparent differences [35], [37].

### Perspectives for Neuroprotective Strategies

In total, results from the present study suggest that oxaliplatin preferentially affects voltage-gated Na^+^ currents with transient inactivation kinetics. What then are the implications of these findings for the design of neuroprotective strategies? Many local anesthestics and antiepileptic drugs target Na^+^ channel properties. While the effects of oxaliplatin have been antagonised *in vitro* by the antiepileptic drug carbamazepine [7], [8], trials in oxaliplatin-treated patients have met with mixed success [6], [38]. Carbamazepine affects both transient and persistent Na^+^ currents and is thought to modulate channel inactivation, with a much higher affinity for inactivated channels [39]. However, it is possible that an alternative mechanism of Na^+^ channel antagonism will be required to avert the development of neuropathy, such as enhancement of slow channel inactivation rather than interaction with fast inactivation properties, as may be observed with the novel antiepileptic drug lacosamide [40]. The development of more sensitive Na^+^ channel modulators should inevitably provide improved selective subtype and current type modulation of channel function, which may prove useful in treating oxaliplatin-induced neurotoxicity, as well as a variety of inherited and acquired Na^+^ channelopathies.
